# 328. One-Day Holiday: An Approach to Managing Daptomycin-Induced CK Elevations in OPAT

**DOI:** 10.1093/ofid/ofad500.399

**Published:** 2023-11-27

**Authors:** Julia B Fabricio, Michael Swartwood, Teresa M Oosterwyk, Asher J Schranz, Claire E Farel, Nikolaos Mavrogiorgos, M C Bowman, Alan C Kinlaw, Renae Boerneke

**Affiliations:** University of North Carolina Medical Center, Durham, North Carolina; University of North Carolina Medical Center, Durham, North Carolina; UNC Medical Center, Chapel Hill, North Carolina; University of North Carolina, Chapel Hill, NC; UNC Chapel Hill, Chapel Hill, North Carolina; University of North Carolina Medical Center, Durham, North Carolina; UNC Medical Center, Chapel Hill, North Carolina; University of North Carolina Eshelman School of Pharmacy, Chapel Hill, NC 27599-7573, NC; University of North Carolina at Chapel Hill Medical Center, Chapel Hill, North Carolina

## Abstract

**Background:**

Daptomycin can be ideal for outpatient parenteral antimicrobial therapy (OPAT) due to its once-daily dosing, tolerability, and lack of nephrotoxicity compared to vancomycin. Though infrequent, skeletal myopathy with associated elevation of serum creatinine kinase (CK) is a clinically significant adverse effect associated with daptomycin. Risk factors associated with CK elevations include initial dose, obesity, chronic kidney disease, and statin use.

Based on a case series by Burdette et. al, the UNC OPAT Program manages asymptomatic mild-to-moderate CK elevations by either a one-day dose holiday with continuation of the same dose or one-day daptomycin holiday followed by dose reduction. We assessed the effectiveness of this strategy for allowing continuation of daptomycin in our patient population.

**Figure 1:**
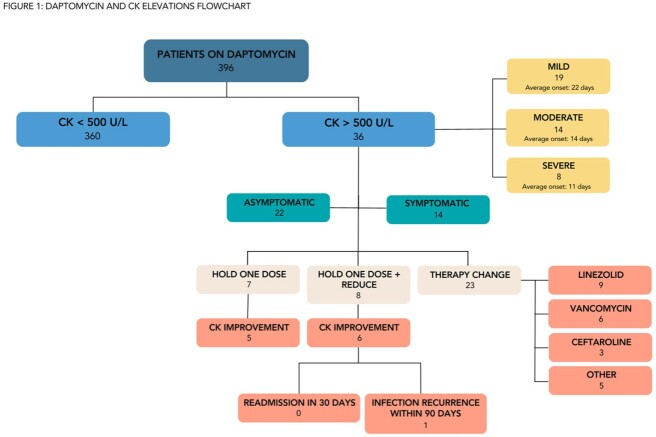
Daptomycin and CK Elevations Flowchart

**Table 1:**
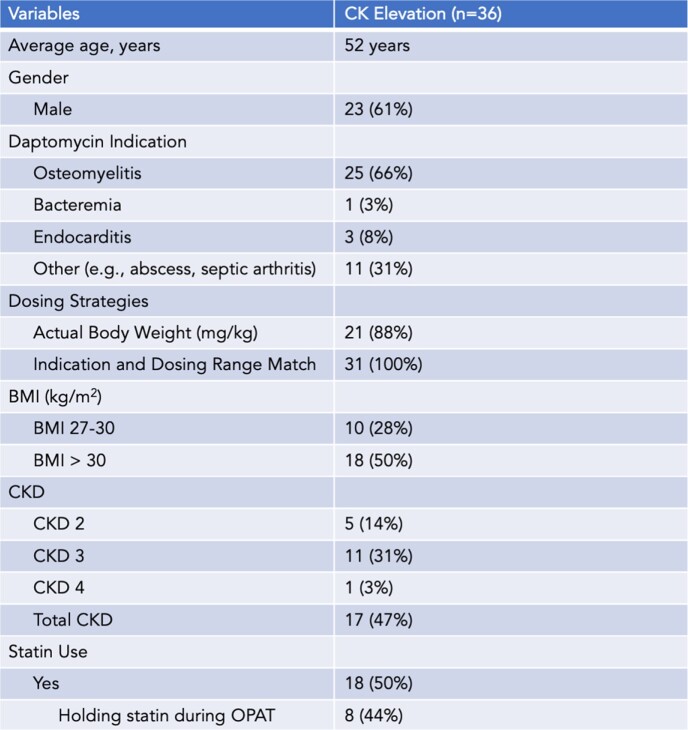
Baseline Demographics

**Methods:**

A total of 396 patients received daptomycin in the OPAT Program from March 2015 to January 2023. We analyzed associated baseline risk factors for patients with CK elevations above 500 U/L and management strategies (e.g., one-day holiday, dose reduction, and/or drug discontinuation). CK elevations were defined as mild (500-999 U/L), moderate (1000-1999 U/L), or severe (≥ 2000 U/L).

**Results:**

Of the 396 patients on daptomycin, 36 (9%) experienced a CK elevation > 500 U/L. 34 (94%) patients required further management. 58% of patients were asymptomatic, with 55% having mild CK elevation. Most reported symptoms were muscle/joint pain. On average, CK elevations occurred 18 days (± 9) into therapy, with a shorter time to CK elevation for severe (11 days ± 2) and moderate elevation (14 days ± 6). Lab abnormalities were managed within one day. Of the 7 patients that had a one-day holiday only, 5 experienced CK improvement. Of the 8 patients with a one-day holiday + dose reduction, 6 showed CK improvement. No patient with dose reductions had readmission during treatment course and one had infection recurrence within 90 days.

**Conclusion:**

The one-day holiday +/- dose reduction adjustment strategy for management of CK elevations allowed for continuation of daptomycin without compromising treatment or leading to hospital readmission. Timely evaluation of safety labs and dose adjustment strategies are key in managing CK elevations for OPAT patients receiving daptomycin.

**Disclosures:**

**Asher J. Schranz, MD, MPH**, WoltersKluwer: Honoraria **Alan C. Kinlaw, PhD**, Genentech: Grant/Research Support

